# Empowering US healthcare delivery organizations: Cultivating a community of practice to harness AI and advance health equity

**DOI:** 10.1371/journal.pdig.0000513

**Published:** 2024-06-06

**Authors:** Mark P. Sendak, Jee Young Kim, Alifia Hasan, Will Ratliff, Mark A. Lifson, Manesh Patel, Iniouluwa Deborah Raji, Ajai Sehgal, Keo Shaw, Danny Tobey, Alexandra Valladares, David E. Vidal, Suresh Balu

**Affiliations:** 1 Duke Institute for Health Innovation, Duke Health, Durham, North Carolina, United States of America; 2 Center for Digital Health, Mayo Clinic, Rochester, Minnesota, United States of America; 3 Division of Cardiology, Duke Health, Durham, North Carolina, United States of America; 4 Department of Electrical Engineering and Computer Science, UC Berkeley, Berkeley, California, United States of America; 5 DLA Piper, Charlotte, North Carolina, United States of America; 6 DLA Piper, Dallas, Texas, United States of America; 7 Community Representative, Durham, North Carolina, United States of America; CSL Behring / Swiss Institute for Translational and Entrepreneurial Medicine (SITEM), SWITZERLAND

## Abstract

Healthcare delivery organizations (HDOs) in the US must contend with the potential for AI to worsen health inequities. But there is no standard set of procedures for HDOs to adopt to navigate these challenges. There is an urgent need for HDOs to present a unified approach to proactively address the potential for AI to worsen health inequities. Amidst this background, Health AI Partnership (HAIP) launched a community of practice to convene stakeholders from across HDOs to tackle challenges related to the use of AI. On February 15, 2023, HAIP hosted an inaugural workshop focused on the question, “Our health care delivery setting is considering adopting a new solution that uses AI. How do we assess the potential future impact on health inequities?” This topic emerged as a common challenge faced by all HDOs participating in HAIP. The workshop had 2 main goals. First, we wanted to ensure participants could talk openly without reservations about challenging topics such as health equity. The second goal was to develop an actionable, generalizable framework that could be immediately put into practice. The workshop engaged 77 participants with 100% representation from all 10 HDOs and invited ecosystem partners. In an accompanying Research Article, we share the Health Equity Across the AI Lifecycle (HEAAL) framework. We invite and encourage HDOs to test the HEAAL framework internally and share feedback so that we can continue to refine and maintain the set of procedures. The HEAAL framework reveals the challenges associated with rigorously assessing the potential for AI to worsen health inequities. Significant investment in personnel, capabilities, and data infrastructure is required, and the level of investment needed could be beyond reach for most HDOs. We look forward to expanding our community of practice to assist HDOs around the world.

## Introduction

Healthcare delivery organizations in the US are facing significant pressure from government agencies to contend with the potential for AI to worsen health inequities. The Health and Human Services Office of Civil Rights finalized a rule in April 2024 to hold healthcare delivery organizations legally liable for discrimination that results from the use of AI [1]. Weeks after the rule was originally proposed, the Attorney General of California launched an inquiry into bias in healthcare algorithms by sending letters to hospital CEOs across the state requesting information about how they address bias in commercial AI tools [2].

Unfortunately, there is no standard set of procedures for healthcare delivery organizations to adopt to navigate these challenges. The assessment of bias in AI is inconsistent and healthcare delivery organizations often lack the necessary personnel and processes to evaluate AI across various domains, leading to inadequate quality control and governance of AI [3]. Furthermore, updates to regulatory guidance to assess bias in AI are piecemeal and incremental, leaving gaps for healthcare organizations to fill and adapt to their unique circumstances [4–7]. Variable documentation and fragmented translation of AI within healthcare organizations may also exacerbate health inequities, favoring high-resource environments and patient populations able to navigate around barriers to care [8]. There is an urgent need for healthcare delivery organizations to present a unified approach to proactively address the potential for AI to worsen health inequities.

### Opportunity for a new community of practice

Amidst this background, Health AI Partnership (HAIP) launched a community of practice to convene stakeholders from across care delivery settings and US geographies to tackle challenges related to the use of AI, starting with the potential for AI to propagate or worsen health inequities.

Healthcare is filled with inspiring examples of communities of practice. Consider scenarios when a best practice is clearly defined, but implementation of the best practice requires specialized expertise that is scarce in low-resource settings. Communities of practice help to bridge the expertise gap. Telestroke programs extend expertise from stroke centers to rural regions [9], Project ECHO extends specialized expertise to support chronic disease management in rural areas and prisons [10], and antimicrobial stewardship outreach networks support specialized programs in small hospitals without relevant expertise [11]. On the other hand, consider scenarios when a best practice is not clearly defined, and experiences from across organizations need to be synthesized to develop an optimal and scalable approach. Once again, communities of practice help bridge this gap. Specialty societies ranging from the American Board of Family Medicine [12], American College of Surgeons [13], American College of Cardiology [14], and Society of Thoracic Surgeons [15] all compile national registries to help healthcare delivery organizations improve and monitor quality of care. Despite numerous related efforts summarized in **Table 1**, a community of practice had yet to form for supporting healthcare delivery organizations to safely, effectively, and equitably harness AI.

**Table 1:** Organizations and collaboratives that develop content about or convene stakeholders around topics related to AI in healthcare.

			Content/Convenings					Target Audience				Business Model					Cross-Sector Activities		
Organization	Description	Website	Academic Output	Best Practice Development	Live Updates	Virtual, Interactive Public Events	Public In-Person Conference	Academic	Government	AI Product Developers	Healthcare Delivery Organizations	Payment for Content	Payment for Convenings	Payment for Participation	Payment for Certification	Structure	Industry Participation	FDA Collaborative Community	Policy Advocacy
AI Healthcare Coalition	An industry advocacy group to influence on health care AI policy and law.	https://ai-coalition.org/	NO	NO	NO	NO	NO	NO	YES	YES	NO	NO	NO	Unclear	NO	Unclear	YES	NO	YES
Alliance for AI in Healthcare	An international multi-stakeholder membership-based advocacy group organized to influence regulatory principles for development and implementation of AI in healthcare.	https://www.theaaih.org/	NO	NO	NO	NO	NO	NO	YES	YES	NO	NO	YES	YES	NO	Unclear	YES	NO	YES
American Medical Informatics Association (AMIA)	A society for health informatics professionals that offers education, training, accreditation, and certifications.	https://amia.org/	YES	YES	NO	NO	YES	YES	YES	YES	YES	YES	YES	YES	YES	501c3	YES	NO	YES
Association for Health Learning and Inference	Premier academic conference for publishing and dissemination of scientific work. Focused on methodological advances and evaluations of AI in healthcare.	https://ahli.cc/	YES	NO	NO	NO	YES	YES	NO	NO	NO	NO	YES	YES	NO	Nonprofit—unspecified	YES	NO	NO
Coalition for Health AI	A community of academic health systems, industry stakeholders, and ecosystem partners developing “guidelines and guardrails” to drive high-quality health care by promoting the adoption of credible, fair, and transparent health AI systems.	https://www.coalitionforhealthai.org/	YES	YES	NO	NO	NO	YES	YES	YES	YES	NO	NO	YES	NO	501c6	YES	NO	YES
Collaborative Community on Ophthalmologic Imaging	A collaborative of academic institutions, government agencies, private businesses, and professional organizations dedicated to establishing standards of practice for innovative ophthalmic imaging.	https://cc-oi.org/	YES	YES	NO	NO	YES	NO	YES	YES	NO	NO	YES	YES	NO	501c3	YES	YES	YES
Connected Health Initiative	A multi stakeholder coalition that advocates for policies and laws related to AI in healthcare. They educate regulators and lawmakers and publish white papers that define industry best practices.	https://connectedhi.com/	NO	YES	NO	NO	NO	NO	YES	YES	YES	NO	YES	YES	NO	Unclear	YES	NO	YES
Digital Health Collaborative	The Digital Health Collaborative is a group of leading healthcare and consumer organizations that share a commitment to “raising the bar” for evidence and value in digital health technology. Through research, grant funding, and regular convenings, the Collaborative advances the development and scaling of evidence-based, efficient, and equitable digital health solutions to improve outcomes and lower costs.	https://phti.com/digital-health-collaborative/	NO	NO	NO	NO	NO	NO	YES	YES	YES	NO	NO	YES	NO	Peter G Peterson Foundation (501c3) program	YES	NO	YES
Health AI Partnership (HAIP)	A multi-stakeholder collaborative who seeks to empower healthcare organizations to use AI safely, effectively, and equitably. Vision is to be the trust partner and up-to-date source of actionable guidance for healthcare professionals using AI.	https://healthaipartnership.org/	YES	YES	YES	YES	NO	NO	NO	NO	YES	NO	NO	NO	NO	Duke program	NO	NO	NO
Healthcare Products Collaborative	Promotes discussion and innovation in the healthcare products community, bringing together regulators, professionals, academics, and thought leaders to tackle industry challenges.	https://healthcareproducts.org/	NO	YES	NO	NO	YES	NO	YES	YES	YES	NO	YES	NO	NO	AFDO and RAPS program	YES	YES	YES
HIMSS (Healthcare Information and Management Systems Society)	A member-based society that covers a large part of health technology ecosystem. This society offers educational resources such as course materials, guides, webinars, and certifications on a range of health information and technology subjects.	https://www.himss.org/	NO	YES	NO	YES	YES	NO	YES	YES	YES	YES	YES	YES	YES	501c6	YES	NO	YES
HLTH	Community for innovators in the healthcare ecosystem. Has a heavy industry focus. Hosts conferences and creates digital content like webinars, podcasts, and blogs.	https://www.hlth.com/	NO	NO	NO	NO	YES	NO	NO	YES	YES	NO	YES	YES	NO	For Profit	YES	NO	NO
KLAS Research	A consulting services that evaluates digital products by aggregating and synthesizing feedback about vendor products.	https://klasresearch.com/	NO	YES	YES	NO	YES	NO	NO	YES	YES	YES	YES	YES	NO	For Profit	YES	NO	NO
Machine Learning for Healthcare	Premier academic conference for publishing and dissemination of scientific work. Focused on methodological advances and evaluations of AI in healthcare.	https://www.mlforhc.org/	YES	NO	NO	NO	YES	YES	NO	NO	NO	NO	YES	YES	NO	501c3	YES	NO	NO
National Academies of Medicine AI Code of Conduct	Aimed at providing a guiding framework to ensure that AI algorithms and their application in health, health care, and biomedical science perform accurately, safely, reliably, and ethically in the service of better health for all.	https://nam.edu/programs/value-science-driven-health-care/health-care-artificial-intelligence-code-of-conduct/	NO	YES	NO	NO	NO	YES	YES	YES	YES	NO	NO	NO	NO	NAM Program	YES	NO	NO
Scottsdale Institute	The not-for-profit Scottsdale Institute was born in 1993 as the brainchild of Stan Nelson, recently retired CEO of Detroit’s Henry Ford Hospital (now Henry Ford Health). Stan and fellow Minnesotan Don Wegmiller, who served as CEO at Allina Health, founded SI as an executive organization of leading health systems to share best practices in information technology (IT). Mission is to inspire and convene thought-leading Member health systems and their partners to leverage information and technology to create effective, affordable and equitable healthcare centered on whole person care.	https://scottsdaleinstitute.org/	NO	YES	NO	NO	NO	NO	NO	NO	YES	YES	YES	YES	NO	501c3	YES	NO	NO
Society for Imaging Informatics in Medicine (SIIM)	Healthcare professional organization for those interested in use of informatics in medical imaging.	https://siim.org/	YES	YES	NO	NO	YES	YES	NO	YES	YES	YES	YES	YES	YES	501c3	YES	NO	NO
The AI Alliance	A community of technology creators, developers, and adopters collaborating to advance safe, responsible AI rooted in open innovation.	There’s no home page. Example press releases: https://newsroom.clevelandclinic.org/2023/12/05/cleveland-clinic-founding-member-of-ai-alliance-an-international-community-of-leading-technology-developers-researchers-and-adopters/ https://newsroom.ibm.com/AI-Alliance-Launches-as-an-International-Community-of-Leading-Technology-Developers,-Researchers,-and-Adopters-Collaborating-Together-to-Advance-Open,-Safe,-Responsible-AI	NO	NO	NO	NO	NO	NO	YES	YES	YES	NO	NO	NO	NO	Unclear	YES	NO	NO
The AI Collaborative (Nuance + The Academy)	A peer learning and consulting services to clinical and operational executives who oversee their organization’s investment in AI tools for healthcare.	https://hmacademy.com/ai-collaborative/	NO	YES	NO	NO	NO	NO	NO	YES	YES	YES	YES	YES	NO	For Profit	YES	NO	NO
Trustworthy & Responsible AI Network (TRAIN)	Through collaboration, TRAIN members will help improve the quality and trustworthiness of AI by: - Sharing best practices related to the use of AI in healthcare settings - Enabling registration of AI used for clinical care or clinical operations - Providing tools to enable measurement of outcomes associated with the implementation of AI - Facilitating the development of a federated national AI outcomes registry for organizations to share among themselves.	https://news.microsoft.com/2024/03/11/new-consortium-of-healthcare-leaders-announces-formation-of-trustworthy-responsible-ai-network-train-making-safe-and-fair-ai-accessible-to-every-healthcare-organization/	NO	YES	NO	NO	NO	NO	NO	NO	YES	NO	NO	YES	NO	Microsoft program (For-Profit)	YES	NO	NO
Valid AI	A collaborative community to advance generative AI in a responsible manner to improve health care and research	https://validai.health/	YES	YES	YES	NO	NO	NO	NO	NO	YES	YES	YES	YES	YES	UC Davis and Node.health (501c3) program	YES	NO	NO

### Inaugural workshop

On February 15, 2023, HAIP hosted an inaugural workshop focused on the question, “Our health care delivery setting is considering adopting a new solution that uses AI. How do we assess the potential future impact on health inequities?” This topic emerged as a common challenge faced by all healthcare delivery organizations participating in HAIP. And despite augmenting our interview sample with AI ethics and bias experts, there was not consensus around how to approach that challenge [3].

Potential case studies were identified through interviews with leaders from across sites [3]. NewYork-Presbyterian had recently completed an internal bias assessment of their internally built postpartum depression model [16]. Parkland Center for Clinical Innovation (PCCI) had recently completed development and validation of their internally built KnowThyPatient patient segmentation model to inform service design [17]. Notably, PCCI focuses on developing and implementing AI solutions within safety net settings that care for historically marginalized populations. Leaders from both organizations agreed to share their case studies with the HAIP community. To help case study teams prepare for the workshop, a Duke Institute for Health Innovation (DIHI) team developed an example case study using a pediatric sepsis prediction model.

The workshop had 2 main goals. First, we wanted to ensure participants could talk openly without reservations about challenging topics such as health equity. To facilitate this, we established 4 community norms. First, the meeting would be confidential with the expectation that learnings would be shared and advanced, but individual statements would not be attributed to participants or organizations. Second, the meeting was an opportunity for all participants to learn from each other with the expectation that there was no known correct approach. We would all gather to develop a generalizable framework for use in clinical practice. Third, participants needed to commit to mutual respect and to provide a safe space for all to raise, explore, and opine on sensitive and challenging issues. And lastly, participants acknowledged and expressed gratitude to the case-study teams for allowing the use of their real-world experiences to surface challenges and opportunities. Critiques of the case studies had to be constructive. In addition to community norms, we restricted workshop participants to HAIP organizations and did not include regulators, journalists, or AI software vendors.

The second goal was to develop an actionable, generalizable framework that could be immediately put into practice. To facilitate this, we complemented participants from HAIP organizations with a team of framework developers and expert discussants. None of the framework developers or expert discussants were involved in either case study. The team of 6 framework developers included a clinician, sociotechnical researcher, computer scientist, lawyer, implementation manager, and community representative. Expert discussants were identified based on familiarity with challenges associated with each case study. For example, a maternal–fetal medicine specialist with AI expertise served as an expert discussant for the NYP algorithm and an epidemiologist with AI expertise served as an expert discussant for the PCCI algorithm.

After each case study presentation, participants entered breakout teams of up to 8 to discuss approaches to the question “Our health care delivery setting is considering adopting a new solution that uses AI. How do we assess the potential future impact on health inequities?” Breakout teams presented their responses for the specific AI solution relevant to each case study, followed by structured comments from expert discussant panels. Finally, each case study team was invited to close out the discussion. A detailed description of the agenda, expert discussants, and framework developers is presented in S1 File. Throughout the workshop, everyone was instructed to practice divergent thinking to highlight approaches or perspectives beyond what had already been discussed.

### Outcomes

The workshop engaged 77 participants with 100% representation from all 10 healthcare delivery organizations and invited ecosystem partners (American Medical Association, DLA Piper, Gordon and Betty Moore Foundation, UC Berkeley). Thirty participants (39.0%) completed a post-workshop survey. On a 5-point scale (1 = *not at all*, 5 = *very much*), participants reported that they were satisfied with the workshop (*mean* = 4.40), felt safe to share their experiences (*mean* = 4.63), found the workshop unique compared to other workshops (*mean* = 4.22), were interested in attending future workshops (*mean* = 4.71), and recommending them to colleagues (*mean* = 4.64). Most importantly, case study presenters rated safeness at 5. In free-text feedback, participants were very pleased with the diversity and mix of perspectives represented as well as the energizing and thoughtful discussion.

### Future directions

In an accompanying Research Article, we share the Health Equity Across the AI Lifecycle (HEAAL) framework and in the coming months, we will curate novel analyses conducted by healthcare delivery organizations applying the HEAAL framework to internally developed or procured AI solutions [18]. On one hand, these articles will demonstrate how organizations can practically apply a standard set of procedures to ensure the safe, efficient, and equitable use of AI solutions. We invite and encourage healthcare delivery organizations to test the HEAAL framework internally and share feedback so that we can continue to refine and maintain the set of procedures.

On the other hand, our research will highlight the challenges associated with rigorously assessing the potential for AI to worsen health inequities. Thoughtful investment in personnel, capabilities, and data infrastructure is required, and the level of investment needed could be beyond reach for most healthcare delivery organizations. While we are excited to cultivate this community of practice, healthcare delivery organizations cannot address health AI equity assessment challenges on their own. We look forward to robust dialogue and action on public–private partnerships to prevent harm due to AI and maximize the benefits to societies around the world.

## Supporting information

S1 File The supplemental information includes a detailed description of the workshop agenda, expert discussants, and framework developers. (PDF)
