# Supplementary material for: Empowering US healthcare delivery organizations: Cultivating a community of practice to harness AI and advance health equity
Source: PLOS Digit Health. 2024 Jun 6;3(6):e0000513. doi: 10.1371/journal.pdig.0000513 (PMC11156347; doi:10.1371/journal.pdig.0000513)
Supplement: S1 File — (PDF) [file pdig.0000513.s001.pdf]

The Health AI Partnership is excited to invite you to the inaugural (virtual) case-based workshop. The workshop will bring together clinical, technical, and operational leaders from health systems across the country to consider the following scenario:

***Our health system is considering adopting a new solution that uses AI.***

***How do we assess the potential future impact on health inequities?***

*Wednesday, February 15, 2023, 1 pm – 5 pm EST*

## *PROGRAM*

1:00 – 1:05 Log in, opening remarks.

1:05 -1:10 Present plans for the afternoon: Establish ground rules to make this a safe space for discussion. Introduce the flow of the workshop.

### Case Study 1: NYP Postpartum Depression

1:10 – 1:20 **Case Presentation:** The case team presents background, initial analyses, sets up health system leadership decisions and discussion questions

1:20 – 1:50 **Breakout Groups:** Break out in 6 workgroups and discuss questions and come up with potential solutions

1:50 – 2:20 **Report Back:** Each workgroup presents their approach for 2 min. All participants discuss for 15 mins

2:20 – 2:35 **Expert panel review:** Each expert shares structured comments, reflects on group presentations, facilitated Q&A

2:35 – 2:45 **Present Solution:** The case team presents an approach to questions and learnings and reflects on how to improve organizational decision making the case

2:45– 3:00 Break /informal discussion

### Case study 2: PCCI Know thy Patient

3:00 – 3:10 **Case Presentation**: The case team presents background, initial analyses, sets up health system leadership decision and discussion questions

3:10 – 3:40 **Breakout Groups:** Break out in 6 workgroups and discuss questions and come up with potential solutions

3:40 – 4:10 **Report Back:** Each workgroup presents their approach for 2 min. All participants discuss for 15 mins

4:10 – 4:25 **Expert panel review:** Each expert shares structured comments, reflects on group presentations, facilitated Q&A

4:25 – 4:35 **Present Solution:** The case team presents an approach to questions and learnings and reflects on how to improve organizational decision making the case

4:35 – 4:45 Break /informal discussion

4:45 – 5:00 **Framework Developers:** Present a draft framework that applies to both cases and similar types of models, discuss follow-up expectations, and closing remarks.

## GROUND RULES AND WORKSHOP LOGISTICS

**Ground rules of the workshop:**

1. We commit to confidentiality. We can share learnings generated in this workshop, but individual statements will not be attributed to participants or organizations.
2. We are on a learning journey together. We are here to work together to develop a generalizable framework we can all use in practice.
3. We commit to mutual respect. This workshop provides a safe space for all participants to discuss sensitive and challenging issues.
4. We are grateful to the case presentation teams. We use real-world experiences to surface challenges and opportunities and not to critique the work of participants or organizations.

**Workshop Logistics:**

The zoom link to join the workshop will be updated on the calendar invite as well as emailed to all the guests.

Please email the Duke Institute for Health Innovation (DIHI) team with any issues: alifia.hasan@duke.edu and mark.sendak@duke.edu

During the breakout discussions, workshop participants are expected to join pre-assigned breakout groups and work on activities/prompts assigned by the case study presenters. The breakout groups are then expected to share their reflections during the case study report back.

Workshop observers will be assigned to a separate room during the breakout session and are not expected to share their reflections during the case study report back.

## CASE STUDY 1 PRESENTER

**Case 1 - Postpartum Depression Case**: A machine learning framework for postpartum depression (PPD) risk prediction using data extracted from electronic health records (EHRs).

**Case background:** Review the paper describing the PPD risk prediction tool here.

**Yiye Zhang, Ph.D., MS**
Dr. Zhang directs the MS program in Health Informatics and teaches data management at Cornell University. She is also the Informatics Director of Clinical Decision Support at NewYork-Presbyterian Hospital, working on implementing prediction models into the electronic health record system. Her research interests are "learning health systems," where data-driven approaches such as machine learning and data mining are applied in creating the next generation of healthcare information technology to better assist healthcare providers and patients in making decisions.

**Ashley N Beecy, MD**
Dr. Beecy is an Assistant Professor of Medicine in the Department of Medicine, Division of Cardiology at Weill Cornell Medicine. She serves as the Medical Director of AI Operations at NewYork-Presbyterian, where she is responsible for the governance, evaluation and implementation of clinical algorithms. She has a research focus in digital health, including the implementation of AI in health care and cardiovascular imaging.

## *CASE STUDY 1 EXPERT DISCUSSANTS*

We have invited a panel of experts to observe the case study and breakout group presentations. These experts will share structured comments on the case and will participate in a facilitated Q&A discussion.

**Melissa McCradden, PhD, M.HSc.**

Dr. McCradden is a Bioethicist with the Department of Bioethics at The Hospital for Sick Children (SickKids), providing clinical and organization ethics consultations, policy, education, and research at SickKids. She is the John and Melinda Thompson Director of Artificial Intelligence in Medicine (AIM) where, as the Integration Lead for AIM, she provides organizational guidance on responsible integration of clinical AI systems impacting patient care.

**Melissa S. Wong, MD**

Dr. Melissa Wong is fellow in Maternal-Fetal Medicine at Cedars-Sinai Medical Center. She earned her medical doctorate at the University of Texas Southwestern Medical Center and completed her residency in obstetrics and gynecology at the University of Chicago Medical Center, where she served as chief resident during her fourth year. Pursuant to her passion for medical education, she completed a medical education fellowship program at UCLA as well as training nationally in the APGO Scholars and Leaders program. After working on faculty in resident education at Cedars-Sinai Medical Center, she resumed pursuing her own educational interests, this time fellowship training in Maternal-Fetal Medicine and a concurrent Masters in Health Delivery Science.

**Jenna Burrell, PhD**

Dr. Burrell is Data & Society's Director of Research. She oversees all aspects of our research program at Data & Society, ensuring the rigor and integrity of our work. Before joining D&S she was a professor at the School of Information at UC Berkeley. Her research focuses on how marginalized communities adapt digital technologies to meet their needs and to pursue their goals and ideals.

## CASE STUDY 2 PRESENTERS

**Case 2 – Know Thy Patient**: A novel approach and method for patient segmentation and clustering using machine learning to develop holistic, patient-centered programs and treatment plans.

**Case background:** Review the paper describing the know thy patient tool here.

**Yusuf Talha Tamer, PhD**
Dr. Tamer is Principal Data and Applied Scientist at PCCI. He has a Ph.D. Computational & Systems Biology and Molecular Biophysics in The UT Southwestern Medical Center at Dallas. His Ph.D. work includes but is not limited to analyzing, understanding, and visualizing experimentally produced big data (e.g., time series, DNA sequence data, market analysis).

**Russell "Rusty" Lewis, Chief Digital Officer**
Mr. Lewis is the Chief Digital Officer for PCCI. Rusty is an innovative technology executive with extensive health informatics, artificial intelligence, analytics, and business experience spanning early stage to Fortune 100 companies. Rusty has successfully led teams ranging in size from 10 to 2000 and authored and edited multiple HIMSS books on the impact of technology on patient care. He holds over 25 US and International patents.

**Steve Miff, Ph.D., President and CEO**
Dr. Steve Miff is the President and CEO of Parkland Center for Clinical Innovation (PCCI), Steve and his team focuses on leveraging technology, data science, and clinical expertise to obtain unique social-determinants-of-health data and incorporate those holistic, personal insights into point-of-care interventions.
Steve earned his Ph.D. and MS degrees in biomedical engineering and a BA in economics from Northwestern University. He has been an adjunct professor of biomedical engineering for more than five years and has authored more than 100 thought leadership, white papers, and peer-reviewed publications.

## *CASE STUDY 2 EXPERT DISCUSSANTS*

We have invited a panel of experts to observe the case study and breakout group presentations. These experts will share structured comments on the case and will participate in a facilitated Q&A discussion.

**Ray Williams, JD**
Raymond Williams is nationally recognized for his experience in litigation and diversity and inclusion. Ray has first-chair jury trial experience, as well as extensive pre-trial litigation experience in complex litigation with a concentration in multi-district litigation where he often serves as national coordination counsel. Ray also has significant experience in a range of internal investigation, compliance, and regulatory matters, particularly within the life sciences and media, sports and entertainment sectors.

**Julia Marcus, PhD, MPH**
Dr. Marcus is an infectious disease epidemiologist and Associate Professor in the Department of Population Medicine at Harvard Medical School and Harvard Pilgrim Health Care Institute, and Adjunct Faculty at The Fenway Institute. Her research focuses on improving the implementation of preexposure prophylaxis (PrEP) to prevent new HIV infections and promote sexual health in the U.S. She has used electronic health record, survey, and qualitative data to identify patients who may benefit from PrEP, evaluate clinical outcomes among PrEP users in real-world healthcare settings, and identify gaps and inequities in the PrEP continuum of care. Her studies are now cited by the Centers for Disease Control and Prevention as key evidence of the effectiveness of PrEP. Her research has also focused on hepatitis C infection, leading to the elimination of national race-based treatment guidelines that had restricted the use of shorter courses of hepatitis C treatment for Black patients.

**Deirdre K Mulligan, J.D**.
Deirdre K. Mulligan is a professor of law at the UC Berkeley School of Information and a Faculty Director of the Berkeley Center for Law and Technology. She was the founding director of the Samuelson Clinic, which she led from 2001-2008. Before coming to Berkeley, she was staff counsel at the Center for Democracy & Technology in Washington. Professor Mulligan's current research agenda focuses on information privacy and security. Current projects include qualitative interviews to understand the institutionalization and management of privacy within corporate America, and role of law in corporate information security policy and practice. Other areas of current research include digital rights management technology and privacy and security issues in sensor networks and visual surveillance systems, and alternative legal strategies to advance network security.

## *FRAMEWORK DEVELOPERS*

We have invited an interdisciplinary team of framework developers to propose a framework for Health AI Partnership health system sites. These individuals are not involved in the case studies and have varied expertise and perspectives.

**Sara Murray, MD, MAS - Clinician Representative**
Dr. Murray is an Associate Professor of Clinical Medicine and serves as Associate Chief Medical Information Officer for Inpatient Care and Data Science at UCSF Health. She also directs the Data Science and Innovation (DSI) team, which uses data science to understand and address the most pressing issues facing the health system. She has a focus on predictive analytics and artificial intelligence (AI) in healthcare, and her team has built infrastructure and governance processes to ensure the deployment of ethical and robust AI. Her team evaluates commercially available tools and algorithms for trustworthiness before implementation and develops new machine-learning models to address pressing health system problems.

**Kathrine Kellogg, Ph.D. - Sociotechnical Scholar Representative**
Professor Katherine Kellogg is the David J. McGrath Jr Professor of Management and Innovation at the MIT Sloan School of Management. Her research demonstrates how organizations can effectively implement algorithmic technologies by including employees in the technology design process, providing training to give employees the skills they need to work with new technologies, and designing new technologies with employees in mind. Kate's current projects examine the collaborative development and implementation of AI-based technologies for frontline providers in healthcare organizations.

**David Robinson, JD - Legal and Regulatory Representative**
David Robinson is a faculty member at Apple University. His new book, Voices in the Code, tells the story of how one community built a life-and-death algorithm in a relatively inclusive, accountable way. Between 2004 and 2014, a diverse group of patients, surgeons, clinicians, data scientists, public officials and advocates collaborated and compromised to build a new transplant matching algorithm. Robinson argues that this experience holds valuable lessons for the design of other high-stakes systems.

**Alexandra Valladares M.S. - Community Representative**
Alexandra is a community organizer whose passion is to tend to the relationships that underpin underserved communities' social realities and promote health and educational advancement, economic mobility, and other aspects of family wellbeing. She offers support to centers in regions

throughout the US in developing and maintaining partnerships and collaborations to provide culturally and linguistically responsive services and helpful resources for families.

**Harini Suresh, PhD. - Computer Scientist Representative**
Harini is a recent PhD graduate and current postdoc at MIT, where her work focuses on incorporating societal context and broad participation throughout the development, evaluation, and use of machine learning systems. Her research has considered this question in the context of several case studies, including arrhythmia detection, x-ray diagnostics, gender-based violence monitoring, and online content moderation. She is also an enthusiastic proponent of interdisciplinary collaboration for thinking about and addressing societally-relevant impacts of technology.

**Will Ratliff M.B.A.- Implementation Manager Representative**
Will is an Innovation Program Manager at DIHI. He leads the sourcing and execution of DIHI's portfolio of innovation pilot projects via the DIHI annual request for applications (DIHI RFA). He has led 25 RFA projects since joining DIHI in summer 2018, including Duke Health's Sepsis Watch solution. Prior to joining DIHI, Will led various clinical strategy, cost reduction, and electronic health record implementation projects for multi-hospital health systems, as part of his experience working in healthcare consulting. Will earned his undergraduate degrees in Economics and French at Vanderbilt University, and his master's in business administration from Duke University's Fuqua School of Business.
